# Haemodynamic stability following adrenaline intracervical block for major haemorrhage during surgical management of late miscarriage: A case report

**DOI:** 10.1016/j.crwh.2022.e00476

**Published:** 2022-12-17

**Authors:** Kamana Subba, Vinodhan Vyapury, Natasha Wetherall, Philip Toozs-Hobson

**Affiliations:** a Department of Obstetrics and Gynaecology, Birmingham Women's and Children's Hospital, NHS Foundation Trust, United Kingdom; b Department of Anaesthesia, Queen Elizabeth Hospital, Birmingham, United Kingdom; c Department of Haematology, Dorset County Hospital, NHS Foundation Trust, Dorchester, United Kingdom

**Keywords:** Adrenaline, Early pregnancy loss, Haemorrhage, Miscarriage, Medical management

## Abstract

The risk of heavy bleeding after a miscarriage is higher in women undergoing medical management compared with surgical. According to the literature, oxytocin receptor mRNA expression in the myometrium is not well formed during early gestation. Adrenaline may be considered in miscarriage which remains refractory to uterotonics and where bleeding from the placental bed may contribute to haemorrhage, before proceeding to surgical intervention. It is used in various settings to control bleeding in gynaecological procedures. A 34-year-old woman in her third pregnancy presented at 15 + 1 weeks of gestation with an open cervical os and bulging membrane. Within three hours of admission, she passed the fetus but failed to deliver the placenta and continued to bleed. She was taken to theatre for surgical management of miscarriage. The bleeding persisted following suction evacuation and despite the standard dose of oxytocin, and misoprostol uterotonics were given. Because the source of bleeding could be the placental bed, potentially low lying at this stage, a 4.4 ml prefix combination of 12.5 μg/ml adrenaline (1:80,000) and lidocaine (20 mg/ml) was administered as an intracervical block equally at four quadrants at the level of the cervical isthmus. This arrested the bleeding immediately and controlled the bleeding until discharge. This technique has not been described previously, which we believe causes vasoconstriction of the placental bed.

## Highlights

• Adrenaline's safety profile has been studied extensively.
• Adrenaline is affordable and easily available.
• Adrenaline may be considered in cases of miscarriage before proceeding to surgical intervention.
• Adrenaline has a very quick onset of action, and so is useful while waiting for uterotonics to work.
• Maternal morbidity can be avoided with adrenaline.

## 1 Introduction

It is estimated that 23 million miscarriages occur every year worldwide, most in the first 12 weeks, termed early miscarriage [1]. Late miscarriage is defined as a loss after the 12th and before the 24th completed week of pregnancy (13^+0^ weeks to 23^+6^ weeks) and happens in 1–2% of pregnancies [2]. The term ‘retained products of conception’ (RPOC) refers to retention of trophoblastic tissue inside the uterine cavity following childbirth, a medical termination of pregnancy or a miscarriage. It is frequently associated with miscarriage at a more advanced stage of gestation and increases the risk of continuous vaginal bleeding, pelvic pain, and infection.

There are three main options for management of miscarriage [3]: expectant, medical using prostaglandins with possible pre-treatment with mifepristone [4] and surgical treatment using aspiration or dilatation and curettage. However, bleeding can persist despite a combination of medical and surgical management, though this is uncommon. According to the major postpartum haemorrhage guideline from the Royal College of Obstetricians and Gynaecologists (RCOG) [5], many other medications and invasive procedures could be applied to control bleeding from the parturient atonic uterus that are not routinely used for management of haemorrhage secondary to miscarriages due to limited applicability.

This case report concerns a patient whose late miscarriage was complicated by major haemorrhage despite surgical evacuation of RPOC and uterotonics. The continuous bleeding was successfully controlled with adrenaline. The aim of this report is to emphasize the valuable role of such medication before proceeding to a more invasive procedure without losing more time.

## 2 Case Presentation

A 34-year-old woman in her third pregnancy presented at 15 + 1 weeks of gestation with minimal vaginal bleeding. She had previously had two pregnancies through in vitro fertilisation (IVF), but had miscarried at 9 and 13 weeks, respectively; this pregnancy was a spontaneous conception. She was otherwise fit and well. An ultrasound scan confirmed a fetal heartbeat with an open cervical os. Given her obstetric history, the patient wanted to be managed conservatively to give every possible chance. Within 3 h of admission, she passed the fetus but failed to deliver the placenta, and she continued to bleed. Despite receiving 800 μg misoprostol per rectum, she continued to bleed and had an estimated loss of 1000 ml. A decision was made to take her to the theatre for surgical management of miscarriage. She was given a general anaesthetic (GA) with modifications. She was pre-loaded with 500 ml of crystalloid whilst having blood crossmatched before the induction of GA, anticipating a degree of haemodynamic instability. However, no issues were encountered besides the predictable changes in haemodynamic status that arose with anaesthetic agents, which were mitigated with aliquots of vasopressor. Besides routine antibiotic prophylaxis, 1 g of tranexamic acid was also given intravenously.

A suction evacuation was performed using a size 12 rigid Karman cannula under ultrasound guidance. The placenta was removed, and 5 units oxytocin was given intravenously. Gentle curettage confirmed the cavity to be empty and uterus size reduced to about 8 weeks with ongoing uterine bleeding. Uterine perforation was excluded as the procedure was conducted under ultrasound guidance. Intra-operative blood loss was estimated at 700 ml. 40 units oxytocin infusion did not reduce blood loss, nor did 0.5 mg of intramuscular ergometrine. The patient's haemoglobin (Hb) dropped from 126 g/l to 96 g/l in the theatre. Despite stability in her blood pressure, her baseline heart rate had risen by approximately 10 beats/min. This was in keeping with a young patient with depleted oxygen delivery reserves. A standard adult unit of packed red cells was transfused as she had already received 1500mls of crystalloids intravenously. Prolonged bimanual compression was performed but the bleeding continued.

Further help was sought and a 4.4 ml prefix combination of 1:80,000 adrenaline (l2.5 μg/ml) and lidocaine (20 mg/ml) was administered as an intracervical block equally at four quadrants at the level of the cervical isthmus. This arrested the bleeding immediately and controlled the bleeding until discharge. This technique, which we believe causes vasoconstriction of the placental bed, has not been described previously.

## 3 Discussion

Epinephrine, also called adrenaline, is a hormone secreted by the medulla of adrenal glands. Adrenaline is a sympathomimetic catecholamine on both alpha and beta-adrenergic receptors using a G protein-linked second messenger system. It has a greater affinity for beta receptors in small doses, although large doses produce selective action on alpha receptors, where it induces increased vasoconstriction through its action on alpha-1 receptors. It was artificially synthesised in 1904 by Friedrich Stolz [6,7]. It has been routinely used in colposcopy for surgery for several decades. The plasma concentration of catecholamines during cervical cone biopsy was studied in 1984 [8]. It is also extensively used in vaginal reconstruction surgeries [9,10]. Owing to its versatile function, other than in gynaecological procedures, adrenaline is used in various settings to control bleeding in the form of topical applications for adenoidectomy [11], subcutaneous injections in burn surgery, and wound infiltration in osteotomy, endobronchial instillation and various head and neck surgeries. Adrenaline is also one of the most common drugs given during cardiac arrest or anaphylaxis. It can be used in women who are asthmatic, where prostaglandins may be contraindicated. As per the 2006 Joint Ambulance Liaison Committee (JRCALC) guideline, adrenaline is reserved for patients with cardiac arrest, anaphylaxis, life-threatening asthma with failing ventilation and if the patient continues to deteriorate despite nebuliser therapy, with precaution with patients on beta-blockers or tricyclic anti-depressants [12].

Misoprostol, a synthetic prostaglandin E1, inhibits intracellular calcium release and platelet aggregation by P2Y1 receptor activation. So, whilst its mode of action in uterine bleeding involves uterine contraction, its impact on calcium and platelet aggregation may also be significant. In cases of heavy bleeding, as per the RCOG Green Top guideline 52, where four units of red blood cells are transfused, if there are no coagulation results, fresh frozen plasma should be given in liaison with the haematologist [5].

We were unable to find any literature on the use of adrenaline for controlling bleeding in miscarriage or termination of pregnancy. General side-effects of adrenaline are uncommon if given in a safe dose. If used in pregnancy, it may reduce placental perfusion and cause tachycardia, cardiac irregularities, and extrasystoles in a fetus, and it can delay the second stage of labour. However, in this case, this was not relevant. Adrenaline has a rapid onset but a short duration of action, ranging from 2 to 10 min. With a half-life of 5 min. This could mean that the vasoconstrictive effect might wear off relatively quickly, and close monitoring for bleeding would be warranted. However, this short period may be enough for the natural clotting mechanisms to “catch up” and allow time for other medications (in this case, uterotonics) to work. Adrenaline is not licensed for use in arresting bleeding associated with pregnancy loss or termination, nor do any professional bodies recommend it for such use. Alternatives to adrenaline include felypressin, which is also combined with local anaesthetic [13].

According to the Miscarriage Association, the prevalence of early pregnancy loss in the UK is 25%. The risk of heavy bleeding is found to be higher in women who undergo medical management compared with surgical management. Per the RCOG Consent Advice No. 10 [14], the risk of heavy bleeding during surgical management of miscarriage is approximately 1–3 in 1000. The uterine contractility is vital in controlling the haemorrhage secondary to the atony, which is significantly improved after complete expulsion of pregnancy products, particularly in women with incomplete miscarriage or retained products of conception with ongoing bleeding. Besides myometrial tone, the other usual structural pathology for heavy bleeding in pregnancy is due to conditions such as a low-lying placenta, invasive placenta spectrum, molar pregnancy, endometritis, or uterine A-V malformation. Nevertheless, various uterotonics are generally given to help with uterine contractility, such as misoprostol 800–1000 microgram PR/PV or oxytocin 5 units slow infusion during surgical management of miscarriage, i.e., suction evacuation when performed under general anaesthesia.

The evidence suggests that the oxytocin receptor (OTR) mRNA expression in the myometrium increases in late pregnancy compared with preterm (24–36 weeks of gestation), whereas decidual expression is found at a much lower level, does not rise at term [15], and is not well formed during early gestation. Several studies performed in female mammal uterine tissues, including bitches [16], showed that the expression of OTR mRNA increases at both parturition and late pregnancy. However, there is a paucity of evidence looking at the sensitivity and response to hormone therapy in early gestation, when the OTR expression and function of myometrial OTR are believed to be underdeveloped [17]. As such, this explains why synthetic oxytocin may not help in these situations.

Mothers and Babies: Reducing Risk through Audit and Confidential Enquiries (MBRRACE-UK) [18] reports that major haemorrhage is one of the major causes of maternal mortality and morbidity in the UK. The management of haemorrhage during parturition is well established as per the underlying causes, viz., tone, tissue, trauma, and thrombin. However, in case of ongoing haemorrhage after early or mid-trimester miscarriage not responding to uterotonics after removal of the pregnancy products, the use of an intra-uterine balloon for tamponade might not be practicable as the cavity tends to be too small to hold the balloon in place, and it is not feasible to carry on with bimanual compression for a prolonged duration.

The placenta develops in the first few weeks of pregnancy while the corpus luteum supports the pregnancy by producing progesterone until around 8 to 12 weeks of gestation, when the placenta takes over hormone production. The placenta is low positioned in early gestation; however, in over 90% of women with the low-lying placenta in the second trimester, the placenta apparently tends to migrate upwards following the development of the lower uterine segment during the third trimester of pregnancy [19]. This could explain the drop in the incidence of the low-lying placenta from 2% in the second trimester (reported at 1–5%) to 0.4% at the end of pregnancy [20].

As evidenced by the data available, a low-lying placenta is quite common until mid-trimester. Inadequate vasoconstriction on the placental bed due to the insufficient myometrial component on the lower part of the uterus could contribute to heavy bleeding, as in this case. This patient had ongoing bleeding after all products of conception were removed and she was not responding to the standard uterotonics, although she was bleeding intermittently in between bimanual compression. To arrest the bleeding from the placental bed, presumably low lying, in this case, we injected the adrenaline/lidocaine combination intra-cervically, aimed at the lower part of the uterus, after ruling out contraindications for adrenaline. This immediately arrested the bleeding and there was minimal bleeding post-operatively and at discharge from the hospital.

Adrenaline may be considered in cases of miscarriage which remain refractory to uterotonics or at least when more time is required for the uterotonics to start working and where bleeding could have a contribution from the placental bed before proceeding to surgical interventions, which carry their own risks. The benefit of this method is that adrenaline is fast acting, inexpensive and widely available (unlike balloons). We are unaware of any cases of placenta previa where it has been used to arrest bleeding by acting on the musculature of the placental bed, but this could be another indication. This is our early experience, and it is very promising at this stage.

## Contributors

Kamana Subba was involved in patient care, was responsible for the conception of the case report, literature search and literature review, drafted the manuscript and revised the article critically for important intellectual content.

Vinodhan Vyapury was involved in patient care and manuscript revision.

Natasha Wetherall contributed to manuscript revision.

Philip Toozs-Hobson was involved in patient care, pioneering of the technique reported, conception of the case report and editing of the manuscript.

All authors approved the final submitted manuscript.

## Funding

This work received no specific grant from any funding agency in the public, commercial, or not-for-profit sectors.

## Patient consent

Obtained.

## Provenance and peer review

This article was not commissioned and was peer reviewed.

## Declaration of Competing Interest

The authors declare that they have no conflict of interest regarding the publication of this case report.
